# Circulating Mediators of Apoptosis and Inflammation in Aging; Physical Exercise Intervention

**DOI:** 10.3390/ijerph18063165

**Published:** 2021-03-19

**Authors:** Barbara Morawin, Anna Tylutka, Jolanta Chmielowiec, Agnieszka Zembron-Lacny

**Affiliations:** 1 Department of Applied and Clinical Physiology, Collegium Medicum, University of Zielona Gora, 65-417 Zielona Góra, Poland; b.morawin@cm.uz.zgora.pl (B.M.); a.tylutka@cm.uz.zgora.pl (A.T.); 2 Department of Hygiene and Epidemiology, Collegium Medicum, University of Zielona Gora, 65-417 Zielona Góra, Poland; j.chmielowiec@cm.uz.zgora.pl

**Keywords:** apoptosis, cell free DNA, tumor necrosis factor, sarcopenia, Tai-Chi

## Abstract

Sarcopenia is an age-related loss of skeletal muscle mass caused by many cellular mechanisms and also by lifestyle factors such as low daily physical activity. In addition, it has been shown that sarcopenia may be associated with inflammation and cognitive impairment in old age. Regular exercise is key in reducing inflammation and preventing sarcopenia and diseases related to cognitive impairment. The study was designed to assess the impact of exercise training on circulating apoptotic and inflammatory markers of sarcopenia in older adults. Eighty older adults aged 70.5 ± 5.8 years were randomized to the physically active group who participated in a 10-month Tai-Chi training session (TC, *n* = 40) and the control group who participated in health education sessions (HE, *n* = 40). Tai-Chi training caused a significant decrease in fat mass (FM) by 3.02 ± 3.99%, but an increase in appendicular skeletal muscle mass index (ASMI) by 1.76 ± 3.17% and gait speed by 9.07 ± 11.45%. Tai-Chi training elevated the plasma levels of C-reactive protein (CRP), tumor necrosis factor (TNFα), and tumor necrosis receptor factor II (TNFRII), and decreased caspases 8 and 9. Despite the increase in TNFα, apoptosis was not initiated, i.e., the cell-free DNA level did not change in the TC group. The study demonstrated that Tai-Chi training significantly reduced the symptoms of sarcopenia through the changes in body composition and physical performance, and improvements in cytokine-related mechanisms of apoptosis.

## 1. Introduction

Aging of skeletal muscles is associated with qualitative and quantitative changes in the muscle structure and function [1,2]. According to the latest definition, sarcopenia is a progressive and generalized skeletal muscle disorder that is associated with an increased likelihood of adverse outcomes, including falls, fractures, physical disability and mortality [3,4]. Studies show that sarcopenia affects 5–13% of people aged 60–70 and 11–50% of people above 80 years of age [5]. The incidence of sarcopenia depends on gender and population. The studies of Shafiee et al. [6] showed that the incidence of sarcopenia is lower in the Asian population in both women and men compared to the non-Asian population (11% vs. 10% in men and 9 vs. 12% in women). Due to the increasing life expectancy in developed countries, sarcopenia is prognosed to become an even more serious and global health problem and a significant economic burden on healthcare systems [7,8]. Therefore, understanding the mechanisms of sarcopenia is of great importance for the development of effective methods to counteract this phenomenon [5].

Skeletal muscle aging is a process associated with a range of factors such as a reduced number of motor neurons and satellite cells, an impaired skeletal muscle regenerative capacity, hormone deficiencies, chronic inflammation, inadequate nutrition and lack of physical activity [9,10,11,12]. However, the exact molecular and cellular mechanisms underlying the loss of muscle mass in the elderly are still largely unknown [12]. There is an increasing body of evidence of the involvement of inflammatory processes and apoptosis as the main causes of the development of sarcopenia [13,14]. Low-grade systemic inflammation, manifested as a high level of pro-inflammatory cytokines, is associated with muscle mass reduction and weakness [15,16]. Special attention was paid to tumor necrosis factor (TNFα) as a potent molecule that stimulates skeletal muscle proteolysis by activating the ubiquitin-proteasome pathway [17,18] and the extrinsic pathway of apoptosis [15]. Transmission of the death signal through the extrinsic pathway begins with TNFα binding to specific membrane receptors, called death receptors, e.g., tumor necrosis factor receptor (TNFR), which are responsible for transmitting the signal into the cell. After TNFα binds to the TNFR membrane receptor, the death signal is transmitted to the adapter protein, which activates procaspase-8 through the presence of the death domain. Caspase 8 (Cas 8) then activates the executive caspase 3 (Cas 3). The receptor pathway can connect to the mitochondrial pathway via the Bid protein and trigger a cascade of events leading to the activation of procaspase-9, which in turn activates effector procaspases through proteolysis [19]. Apoptotic signaling cascades induce DNA fragmentation and lead to the cell death. Measurements of extracellular DNA fragments, cell-free DNA (cfDNA), allow us to observe the phenomenon of increased apoptosis in aging [20,21].

One of the areas that weakens with age is cognitive decline. Interestingly, one of the ways to improve cognitive function is regular physical activity. Beneficial effects of physical activity on cognitive function depending on the inherent qualities of the particular activity type. According to Ingold et al. [22] the greatest improvement in cognitive function are mainly as a result of a closed activity such as Tai-Chi. Marial arts are known to have a positive effect on cognitive functions because of the need to learn selected movement sequences [22]. Moreover, regular physical activity, including cardiovascular and resistance exercise, has been shown to be associated with lower levels of pro-inflammatory cytokines and higher antioxidant capacity as well as improved functional fitness [23]. However, the available literature concerning sarcopenia has not clearly determined which aspects of age-related changes result from exercise-induced factors and which are caused by a sedentary lifestyle.

Daily physical activity, which affects the muscle tissue in particular, dramatically declines with age. Unfortunately, many forms of exercise that are effective in sarcopenia prevention are reluctantly conducted by the elderly. Therefore, new exercise forms, such as Tai-Chi, are offered as alternative methods to increase or maintain muscle mass and function. Tai-Chi is a full-body exercise that combines slow and rhythmic movements in a continuous sequence in which the center of gravity shifts with the movements of each foot. Tai-Chi improves muscle balance, proprioception, strength and endurance [24]. Numerous studies have investigated Tai-Chi as an intervention for a wide variety of health problems, especially balance control and musculoskeletal diseases [25,26,27,28,29].

The study was designed to evaluate the relationship between lifestyle exercise and sarcopenia and to demonstrate the effectiveness of Tai-Chi training in the reduction of the circulating apoptotic and inflammatory markers compared to inactive older adults.

## 2. Materials and Methods

### 2.1. Subjects

**Figure 1:**
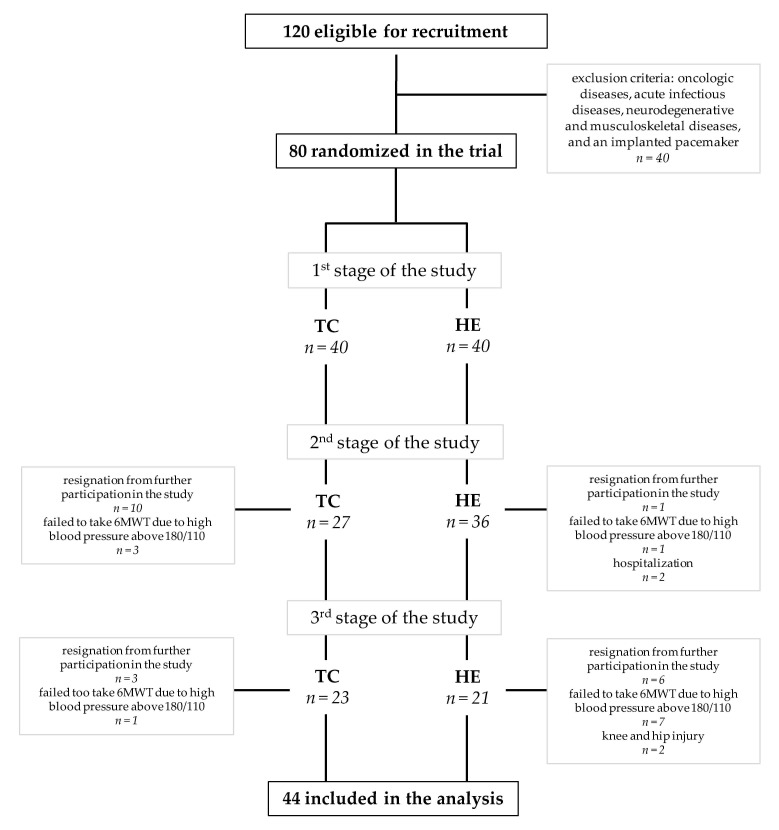
Flow chart of study subjects’ participation. Abbreviations: TC: Tai-Chi training, HE: health education, 6MWT: the 6-min walk test.

A total of 120 subjects were recruited from the University of the Third Age. The University of the Third Age is a teaching institution for the elderly in retirement age. The aim of the measure is to improve the quality of the elderly life. U3A students participate in various classes and courses that create opportunities to constantly expand their knowledge, skills and stimulate social activation. The inclusion criterium was age between 60 and 90 years. Oncologic diseases, acute infectious diseases, neurodegenerative and musculoskeletal diseases and an implanted pacemaker were the exclusion criteria and had to be diagnostically confirmed by a medical specialist and the investigator. The subjects’ current health status and lifestyle were assessed using the health history questionnaire performed during the recruitment stage and in the course of the project [30]. The study included 80 older adults of the University of the Third Age (U3A) (females *n* = 72; males *n* = 8), aged 70.5 ± 5.8 years. On the basis of Community Healthy Activities Model Program for Seniors (CHAMPS) [31], Tai-Chi training was proposed as the most favorite form of physical activity (by approximately 60% of the respondents) as opposed to leisurely walking, water gymnastics or Nordic walking. The subjects were randomly assigned to a Tai-Chi group, who participated in a 10-month training course (TC, *n* = 40), and the control group (HE, *n* = 40), who participated in a health education course. Eventually, 44 subjects completed the Tai-Chi training (*n* = 23) or health education course (*n* = 21) (Figure 1). A written consent for participation was obtained from all U3A students, who were informed of the aim of the study. The study protocol was approved by The Bioethics Commission at Regional Medical Chamber Zielona Gora, Poland (No. 01/66/2017), in accordance with the Helsinki Declaration.

### 2.2. Training and Health Education

**Figure 2:**
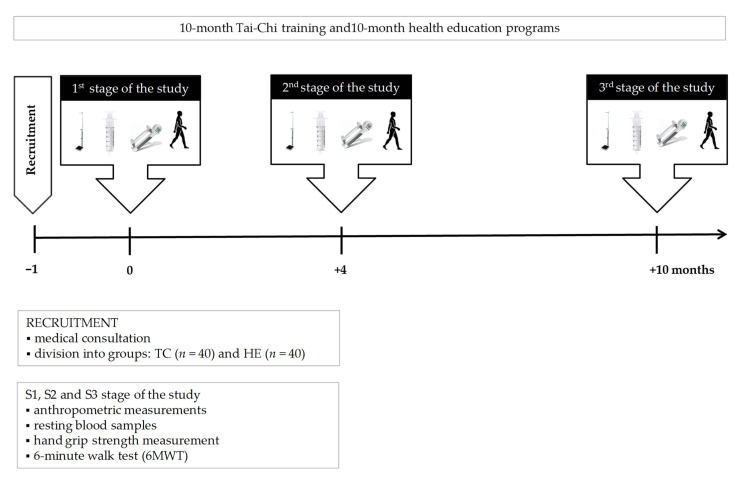
Illustration of the study design. Abbreviations: TC: Tai-Chi training, HE: health education.

The Tai-Chi training sessions and the health education course (TC vs. HE) were conducted in 10-person groups for 10 months. Each TC or HE session lasted 40 min and was held twice a week. Participants in the Tai-Chi group attended a Yang-style 24-form Tai-Chi training program. During Tai-Chi training, the range of exercise elements was increased every month by 2–4 exercises by the Tai-Chi instructor. The health education sessions included lectures and discussions with a physician, a dietician or a psychologist. The biochemical and sarcopenic analysis was performed before (S1; the 1st stage of study), then again after 4 months (S2; the 2nd stage of study) and finally after another 6 months (S3; the 3rd stage of study) of the Tai-Chi training or the health education program (Figure 2).

### 2.3. Analysis of Sarcopenia

The assessment of sarcopenia was carried out in accordance with the algorithm by the European Working Group on Sarcopenia in Older People 2 (EWGSOP2) [3] based on three criteria: reduced muscle mass, reduction of physical performance and reduction of skeletal muscle contraction strength (Table 1).

#### 2.3.1. Body Composition and Muscle Mass

The bioelectrical impedance method was used to estimate body mass (BM), body composition fat-free mass (FFM) and fat mass (FM) by means of Tanita Body Composition Analyzer MC-980 (TANITA, Tokyo, Japan), which, following the manufacturer’s guidelines, was calibrated prior to each test session. Duplicate measures were made with the participant in a standing position and the mean values were used for the final analysis. The measurements were taken between 7:00 and 9:00 a.m., prior to blood sampling, and the recurrence of measurement was 98%. The subjects of the study were advised to fast for at least 12 h before the measurement of body composition. They were asked to avoid heavy physical exertion and to avoid consuming alcohol as well as any other drinks for 3 h before the test. The subjects were supposed to empty their bladder 30 min before the measurement. Moreover, while waiting for the test, the patients were obligated not to move around but stay in a sitting position for 10 min before the test. During the measurements, the subjects were not allowed to have any metal nor cream on their lower and upper limbs. Sarcopenia cut-off points for low muscle quantity were evaluated using the appendicular skeletal muscle mass (ASM kg) and appendicular skeletal muscle mass index (ASMI kg/m^2^) [3,32,33].

#### 2.3.2. Physical Performance

The 6-min walk test (6MWT) was performed following the standards of European Respiratory Society and American Thoracic Society [37]. The aim of the test was to walk as fast and as far as possible over a span of six minutes along a marked 30-m walkway with cones placed at regular intervals to indicate the distance covered. The subjects were allowed to self-pace and to rest as needed. The total distance covered was recorded and the 6MWT gait speed was calculated by the following equation: gait speed (m/s) = total distance(m)/360 s. Following classification by Middelton et al. [38], a gait speed within the range of 1.3 to 1.8 m/s classified the older adults as active, while a gait speed <1.3 m/s classified them as inactive. The cut-off point for sarcopenia was 0.8 m/s.

#### 2.3.3. Grip Strength

The isometric strength of hand grip was measured using a hand dynamometer KERN type MAP130 (KERN & SOHN GMbH, Balingen, Germany). The participants were instructed to squeeze the dynamometer as hard as they could while seated in an upright posture with arms by their sides. The measurement of the strength of the dominant and non-dominant hand was taken twice. The force associated with the maximal trial was documented in kilograms. To determine the grip strength that the subjects should obtain, the cut-off points according to EWGSOP2 were taken into account. Sarcopenia cut-off points for low strength by grip strength are as follows: <16 kg for women <27 kg for men.

### 2.4. Blood Sampling

Blood samples were taken from the median cubital vein using S-Monovette-EDTA K2 tubes (SARSTEDT AG & Co. KG, Nümbrecht, Germany) for morphology and S-Monovette serum tubes (SARSTEDT AG & Co. KG, Nümbrecht, Germany) for the other markers’ determination (Figure 2). The subjects were fasted before blood sampling and were not physically active for at least 48 h before blood collection. Serum samples were left to clot for 45 min before centrifugation and then centrifuged at 3000× *g* and +8 °C for 10 min. Aliquots of serum were stored at −80 °C.

### 2.5. Hematological Variables

Peripheral blood morphology including white blood cells (WBC), red blood cells (RBC), hemoglobin (HB), hematocrit (HCT) and platelets (PLT) were determined by means of 3 diff BM HEM3 Biomaxima (Biomaxima, Lublin, Poland).

### 2.6. Biochemical Markers

Total cholesterol (TChol), high-density lipoprotein (HDL), low-density lipoprotein (LDL) and triglycerides (TG) were determined using BM200 Biomaxima (Biomaxima, Lublin, Poland). The non-HDL cholesterol was calculated by subtracting HDL from the total cholesterol concentration. Spectrophotometer DP 310 Vario II (Diaglobal GmbH, Berlin, Germany) was used for spectrophotometrical measurement of the glucose level. The hsCRP was measured using a high-sensitivity assay in duplicate by means of commercial kit from DRG International (DRG International, Inc., Springfield Township, NJ, USA) with a detection limit of 0.001 mg/L.

### 2.7. Apoptotic and Inflammatory Markers

TNFα level was determined by R & D Systems kits (R&D Systems, Inc., Minneapolis, MN, USA), and its detection limit was 0.038 pg/mL. The soluble tumor necrosis factor receptor I (TNFRI) and soluble tumor necrosis factor receptor II (TNFRII) were identified by an R & D Systems kit (R&D Systems, Inc., Minneapolis, MN, USA), and their detection limits were 1.2 pg/mL and 2.3 pg/mL, respectively. Caspases Cas 8 and Cas 9 were determined by Invitrogen ThermoFisher Scientific kits (Bender MedSystems GmbH, Vienna, Austria), and their detection limits were 0.1 ng/mL and 0.4 ng/mL, respectively. The total circulating fragments of DNA were measured directly in the serum using Quant-iTTM DNA high-sensitivity assay kit by Invitrogen ThermoFisher Scientific (Life Technologies Holdings Corporation, OR, USA) and Qubit 3.0^®^ fluorometer (Life Technologies Holdings Pte Ltd, Singapore) according to the instructions of the manufacturer. All samples were analyzed in duplicate or triplicate in a single assay to avoid inter-assay variability.

### 2.8. Statistical Analysis

Statistical analyses were performed by means of statistical software Statistica 13.1 (StatSoft Inc., Tulsa, OK, USA) and the R system, version R1.4.1103. The assumptions for the use of parametric or non-parametric tests were checked using the Shapiro-Wilk test to evaluate the normality of the distribution. The significant differences in mean values between the groups (TC vs. HE) were assessed by the test t-student. If the normality and homogeneity assumptions were violated, the Mann-Whitney non-parametric test was used. Comparisons of repeated measurements (S1 vs. S2, S1 vs. S3 and S2 vs. S3 test series) were assessed by the two-way ANOVA with repeated measures and Tukey’s HSD test or the Friedman non-parametric test. A mixed ANOVA analysis was performed in R environment to determine the significance of the interaction between the studied independent variables (study group and stages). The percentage changes of the variables analyzed in each study group between the study stage S1 vs. S2 and S1 vs. S3 were calculated as Δ(%):((S2−S1)/S1) × 100 and as Δ(%):((S3−S1)/S1) × 100. In order to describe the relationships between the physical activity level and the inflammatory response Pearson’s correlation coefficients were calculated. Alpha was set to *p* < 0.05 and values less than this were considered significant. The results are expressed as mean and standard deviation (x ± SD).

## 3. Results

### 3.1. Body Composition

The body mass index (BMI) in the TC group ranged from 18.8 to 33.4 kg/m^2^, whereas in the HE group, the BMI amounted to between 21.4 and 32.5 kg/m^2^ (Table 2). Jointly, in both groups, approximately 20% of the study subjects were classified as obese (BMI ≥ 30 kg/m^2^), 52% as overweight (BMI 25.0–29.9) and 27% as normal weight (BMI 18.5–24.9). The BMI value was highly correlated with fat mass content in the TC group (r = 0.879, *p* < 0.0001) and HE group (r = 0.939, *p* < 0.0001). In the subjects with a high percentage of fat mass (FM%), the concentration of hsCRP was detected at >3 mg/L, which confirms the relationship between the increased fat content and the severity of inflammatory processes in the elderly. The 10-month Tai-Chi training resulted in significant changes in body composition (Table 3). BMI was slightly reduced at the 3rd stage of the study, mainly by a decrease in fat mass. FM% decreased by 3.02 ± 3.99% at the 3rd stage compared to the initial level. There was also a significant increase in the muscle mass of the lower limbs in the TC group, while no changes in the muscle mass of the upper limbs were observed.

### 3.2. Muscle Quantity

Approximately 90% of the TC group and 85% of the HE group reached the correct value for ASM according to Cruz-Jentoft et al. [3]. AMSI was recorded at 6.9 ± 0.6 kg/m^2^ in the TC group and at 6.8 ± 0.7 kg/m^2^ in the HE group. The 10-month Tai-Chi training course induced a significant increase in ASM and ASMI compared to the initial level, whereas no changes were observed in the HE group (Table 4).

### 3.3. Physical Performance

The elderly covered the distance of 442.0 ± 77.3 m at a gait speed of 1.23 ± 0.21 m/s during the 6MWT at the 1st stage of the study. Approximately 98% of the subjects achieved a gait speed above the reference value of 0.8 m/s, which determines the cut-off point for sarcopenia according to Cruz-Jentoft et al. [3,34]. Moreover, 86% of the elderly exceeded 1 m/s, which indirectly indicates healthy aging in the students at the U3A [39,40]. A significant increase (~7%) in gait speed was observed in both TC and HE groups at the 2nd stage of the study (Table 4). This increase could result from so-called “learning to walk” that is observed when the 6MWT is re-taken. Such an effect, however, no longer occurs in subsequent tests [41,42]. At the 3rd stage of our study, approximately 70% of the TC subjects reached a gait speed >1.3 m/s, while only 28% of the HE group exceeded 1.3 m/s.

### 3.4. Grip Strength

Reduced muscle strength is regarded as the first criterion in the assessment of sarcopenia [3]; hence, in this study, a potential sarcopenia was already demonstrated in approximately 16% of U3A students. Potential sarcopenia was demonstrated in 4.8% of the subjects in the TC group, while in 25.2% in the HE group. No increased number of subjects with potential sarcopenia was observed at the 2nd and 3rd stage of the study. No sarcopenia was observed in any of the subjects, neither in the TC group nor in the HE group at any stage of the study. After 10 months of Tai-Chi training, no significant changes were recorded in the grip strength of the non-dominant hand when compared to the values at the initial level and in HE group (Table 4). However, significantly reduced muscle strength of the dominant hand was observed in TC group between the 2nd and 3rd stage of the study.

### 3.5. Hematological Variables

In both TC and HE groups, white blood cell count and red blood cells count were found to fall within the referential range, i.e., 4.0–10.2 103/µL for WBC and 4.0–5.5 106/µL for RBC. There were no statistically significant differences in WBC count between TC and HE groups (Table 5). However, the HE group demonstrated a significant decrease in WBC count at the 2nd and the 3rd stage. In the TC group, RBC count did not change during the observation, while in the HE group, a significant reduction was observed between the 2nd and the 3rd stage of the study. The HB concentration did not alter in either of the groups in contrast to HCT, which was found to increase at the 3rd stage of the study compared to the 1st stage in both groups. Platelets (PLT) count was observed to rise significantly in the TC group and to decrease in the HE group at the 3rd stage of study.

### 3.6. Biochemical Markers

The values of TChol, TG as well as LDL and HDL were found to be at similar levels in all subjects. Approximately 85% of all the subjects exceeded the reference values for TChol > 200 mg/dL and 39% of them exceeded the reference values for TG > 150 mg/dL. Low levels of HDL lipoproteins (<60 mg/dL) were found in only 5% of the subjects. The lipoprotein-lipid profile, especially LDL and HDL, were found to change in all subjects within the 10-month study (Table 5). However, it seems unlikely that Tai-Chi training could have such a significant impact on the lipid profile as to increase the risk of the endothelial dysfunction. The observed changes could be related to nutritional factors or hypolipidemic drugs intake. hsCRP concentration fell within reference values (<5 mg/dL) in all the analyzed subjects. Tai-Chi training led to a slight increase in hsCRP level. The hsCRP level was found to exceed the reference values only in 25% of the subjects after 4 months of Tai-Chi training (Table 5). Glucose concentration ranged from 72.3 to 224 mg/dL; however, approximately 16% of the subjects demonstrated the prediabetes level > 115 mg/dL in both groups. Our study showed that Tai-Chi training and health education resulted in a decrease in glucose concentration by 9.33 ± 10.84% (Table 5).

### 3.7. Apoptotic and Inflammatory Markers

Tai-Chi training resulted in an increased concentration of TNFα and TNFRII, but it did not significantly affect the TNFRI level. A tendency towards higher values of TNFα, TNFRI and TNFRII was recorded in the TC group compared to the HE group (Table 6). Tai-Chi training and health education significantly reduced Cas 8 and Cas 9 concentrations at the 2nd and the 3rd stage of the study; however, Cas 8 levels in TC group were found to decrease more considerably (Table 6). These results suggest that, despite the increase in TNFα level, the process of apoptosis was not initiated. The level of cfDNA was not observed to change following Tai-Chi training, whereas the HE group demonstrated a significant increase in cfDNA at the 2nd stage of study compared to the initial values (Table 6).

## 4. Discussion

Sarcopenia increases the risk of falls, leads to mobility disorders and reduces the ability to perform everyday activities, which contributes to a reduced quality of life [3]. In addition, it has been shown that sarcopenia and frailty may be associated with cognitive impairment in old age. The International Consensus Group of the International Academy of Nutrition and Aging and the International Association of Gerontology and Geriatrics proposed a common concept of “cognitive frailty,” which they defined as the simultaneous presence of physical frailty and cognitive impairment. Current research focuses on the relationship between frailty, sarcopenia, cognitive frailty and global functionality [43]. Tamura et al. [44] observed that in almost all people diagnosed with frailty (97%) and people with sarcopenia (90%), cognitive impairment was also diagnosed. The most promising method positive impact on cognitive function throughout life and reduce the risk of age-related cognitive decline is physical activity [45]. Exercise increases neurogenesis, angiogenesis and mitogenesis through neurotrophic factors, and reduces oxidative stress. Regular exercise is crucial in the prevention of diseases associated with impaired cognitive functions and helps to maintain so-called mental wellbeing [23,46].

Due to the increasing incidence of sarcopenia and the decline in cognitive functions in the elderly, the development of preventive and therapeutic strategies is becoming a pressing necessity [47]. Tai-Chi is a type of physical exercise which is practiced mainly in order to improve the body balance, flexibility and strength, and it is easy to understand and perform. Tai-Chi is not only an exercise for the body, but also the mind, as it combines physical and cognitive elements. These exercises include learning movement patterns, attention training and multitasking. Tai-Chi may be an effective intervention slowing down the decline of cognitive functions in the elderly [48], but most of all it affects the mobility of the elderly. The effectiveness of Tai-Chi in the improvement of body composition and functional mobility in the elderly has already been demonstrated in numerous available studies [25,26,27,28,29,49,50,51].

Regular physical activity is essential for healthy aging [52]. Lifestyle exercise protects against various components of weakness, namely, functional and cognitive decline; it improves the quality of life of the elderly and reduces the risk of physical and mental disability. In our research, potential sarcopenia was identified in 16% of all subject. This shows that the U3A students mainly represented healthy aging. After 10 months, no higher percentage of people with confirmed sarcopenia or potential sarcopenia was observed. The U3A students were characterized by a good functional condition, as shown by their results obtained in the 6MWT (442 ± 77 m). Approximately 98% of the elderly achieved a gait speed > 0.8 m/s and a large percentage of the subjects (~34%) obtained a gait speed > 1.3 m/s. The U3A students also achieved high results in the grip strength of the dominant hand. Approximately 85% of the subjects obtained results above the reference value of more than 27 kg for men and 16 kg for women according to Cruz-Jentoft et al. [3].

Numerous studies have shown that exercise has a beneficial effect on the body composition in healthy elderly people [53]. In our study, the 10-month Tai-Chi training resulted in changes to body composition, primarily in fat mass reduction. We also demonstrated an increase in ASM and ASMI at the 3rd stage of study as opposed to the control group. These results show that regular physical activity reduces the content of fat mass and increases muscle mass. In our study, the muscle mass increase referred mainly to the lower limbs, which may result from the specificity of Tai-Chi training. Tai-Chi training also improved the physical fitness of the elderly, which was evidenced by the increase in gait speed and the distance covered in the 6MWT in the TC group. Interestingly, the increased gait speed was recorded in both groups at the 2nd stage of the study, but this may be related to the “learning to walk” effect. According to Sciurba et al. [41] and Wu et al. [42], once the study subjects have become familiar with the 6MWT, the distance they cover may be extended by up to several percent. At the 3rd stage of study, only participants in the TC group increased their gait speed. We observed that approximately 70% of participants in the TC group achieved a gait speed value above 1.3 m/s at the last stage of the study, which proves their high physical fitness level. Similar observations were made by Gow et al. [54], who assessed the gait dynamics in response to training. Some improvement in gait speed and performance was also recorded by You et al. [55], Manor et al. [56] and Zou et al. [57]. It is noteworthy that the 10-month Tai-Chi training did not increase the muscle strength of the upper limbs; on the contrary, at the last stage of study, we noticed a decrease in the strength of the dominant hand, while no significant changes were identified in the control group. Despite the decline in muscle strength in the Tai-Chi group, about 90% of the subjects obtained normal values according to Cruz-Jentoft et al. [3]. The observed lack of increase in the muscle strength of the upper limbs in the TC group may be accounted for by the characteristics of Tai-Chi, which primarily strengthens the muscles of the lower extremities, including the knee extensors [58,59,60] and flexors [61]. This, in turn, favorably affects the balance, dynamic stability and motor control [62], which was also proven by our TC group’s increased performance in the 6MWT after 10 months of training. As in the study by Kumar [63], Tai-Chi exercises were demonstrated to produce better effects in reducing the fear of falls, thereby improving balance control and functional mobility, rather than improving muscle strength, especially in the upper limbs. The increase in muscle strength is mainly observed in resistance training, which is considered an important strategy to prevent muscle wasting. Unlike resistance exercise, aerobic exercise has little effect on strength or muscle mass, but it improves aerobic capacity and metabolic regulation [64]. Yan et al. [65] showed that endurance training could inhibit the apoptotic pathway in skeletal muscles, whereas Ko et al. [66] demonstrated that aerobic exercise controlled the expression of myostatin mRNA. Therefore, in order to counteract sarcopenia, it is recommended to combine both aerobic and resistance exercises.

Physical training prevents the loss of muscle mass in the elderly, but also reduces systemic inflammation. In contrast, there is insufficient evidence about the benefits of exercise in reducing local inflammation in the skeletal muscles in the elderly. Regular resistance training improves resistance to skeletal muscle damage in the elderly, but the process is slower than in young people. The slower repair of damaged skeletal muscles may be due to chronic inflammation. An indirect evidence for the role of inflammation in sarcopenia is the correlation of markers of systemic inflammation with the loss of muscle mass and strength in the elderly [67]. We demonstrated elevated TNFα levels after 10 months of Tai-Chi training and we also observed a similar rise in TNFα in the HE group; however, TNFα tended to reach higher values in the TC group than the HE group. Importantly, the increase in TNFα concentration did not reach considerable values in any of the study subjects. The available studies on the elderly show that regular physical activity reduces the secretion of pro-inflammatory cytokines and increases the release of anti-inflammatory cytokines [68]. A review of randomized controlled trials reported that a training duration in excess of 6 months was required for the reduction of systematic inflammatory markers [69]. Monteiro Junior et al. [68] detected that high levels of TNFα (77.6 ± 17.1 pg/mL) decreased with exercise, while low levels (2.68 ± 4.26 pg/mL) showed no significant changes. It is noteworthy that the initial TNFα values in our TC group were recorded at the level of 0.8 ± 0.4 pg/mL and they increased to the level of 3.8 ± 1.8 pg/mL at the 3rd stage of the study. The increase was less considerable in HE and the differences may be accounted for by TC group adaptation to exercise.

To date, extensive research has been conducted into the impact of Tai-Chi training on older seniors physical and mental functioning. Most researchers analyzed the influence of Tai-Chi training on the elderly with specific diseases. Robins et al. [70] investigated the effect of 8-week Tai-Chi intervention on cardiovascular risk in women aged 48 years and observed a decrease in fatigue. Significant changes, such as decreasing levels of pro-inflammatory cytokines INF-gamma and TNFα, were also recorded 2 months post intervention [70]. On the other hand, Sungkarat et al. [69] analyzed the effect of Tai-Chi training on cognitive functions and plasma biomarkers, e.g., IL-10 and TNFα, in the group aged 67.9 years, and did not record any statistically significant changes in the level of pro-inflammatory cytokines after a 6-month home exercise program. In our research, however, we posed a question which had never been asked before, namely: what is the impact of Tai-Chi training on the aging processes of skeletal muscles?

Sarcopenia is a condition of multifactorial origin, and elevated levels of pro-inflammatory markers play an important role in its development. Nuclear apoptosis is another significant and well-documented factor that contributes to skeletal muscle mass loss. The contribution of the extrinsic apoptotic pathway to skeletal muscle mass loss, especially during the aging process, has been studied less thoroughly than the nuclear apoptosis [71]. TNFα is one of the cytokines capable of inducing apoptosis, and its signaling pathway involves two membrane receptors: TNFRI and TNFRII. Bruunsgaard et al. [72] showed an age-related increase in serum concentration of specific cytokines. The levels of TNFα and IL-6 were reported to be higher in the elderly above 80 years of age than in young people aged 18–30 years [72]. Some researchers also emphasized the association between an increase of circulating cytokines and the sarcopenic process. The effect of TNFα on skeletal muscle is dependent on the differentiated status of the muscle cells and the physiological conditions, but it is also contingent on aging-related adaptation of skeletal muscles [73]. According to Pedersen et al. [74], elevated levels of TNFα are associated with a lower appendicular skeletal muscle mass and reduced grip strength in both elderly men and women [75]. In our study, despite the increase in TNFα after 10 months of Tai-Chi training, no decrease in muscle mass was observed; on the contrary, an increase in ASM was identified. This indicates that the increase in TNFα does not always have a negative impact on muscle mass. However, at the 3rd stage of the study, we recorded an increase in the blood concentration of TNFα in the elderly TC group with a simultaneous decrease in their muscle strength. According to Nicklas and Brinkley [76], TNFα also weakens the power production of myocytes regardless of muscle wasting, while Caldow et al. [14] reported no association between muscle strength and markers of muscle inflammation in the elderly. Contrastingly, Grewie et al. [77] recorded a negative correlation between the muscle strength and inflammatory markers in people over 75 years of age. Caldow et al. [14] suggested that this relationship may increase with age, when skeletal muscle wasting is observed and the ability to perform daily activities decreases. In addition to the detrimental effect of inflammation on sarcopenia, it has also been shown to affect cognition. Especially TNFα activity may impair cognition [78]. It is still unknown which areas of cognitive function may be most affected by inflammation [79]. In many studies, elevated levels of TNFα have been observed in elderly people with cognitive impairment [80]. In our study, the mean values of TNFα and hsCRP did not exceed the reference values of U3A students. Low values of inflammatory indicators may indicate that these people do not have cognitive disorders. In our study, the mean values of TNFα and hsCRP in U3A students did not exceed the reference values, which may indicate that the subjects do not have cognitive impairment. The active lifestyle of the subjects may have an influence on the low values of TNFα and hsCRP. According to Sartori et al. [79], the increase in inflammation with age is likely a consequence of the aging of the immune system, and there are certain lifestyle factors such as exercise and diet that may also play a key role in inflammation.

TNFα can induce apoptosis by interacting with specific TNF receptors (TNFRI and TNFRII). Both receptors have similar extracellular domains but differ structurally in the intracellular domain and only TNFRI contains the death domain. TNFRI mediates signaling of both apoptosis and cell survival unlike TNFRII, which is mainly involved in the latter. Following ligand binding, TNFRI can initiate apoptosis by activating pro-caspase 8, which then activates pro-caspase 3. On the other hand, the formation of a complex between TNFα and the TNFRI receptor may induce an anti-apoptotic response, which involves the transcription factor NF-kB. The presence of the death domain in the internal domain of the TNFRI receptor determines whether activation of the apoptotic or anti-apoptotic pathways will occur [73]. In our study, an increase in the concentration of TNFα and TNFRII receptor was observed in both groups, but at the same time, no changes in the level of TNFRI receptor and a decrease in the concentration of caspase 8 and caspase 9 were detected. Additionally, in physically active elderly people, statistically significantly lower levels of caspase 8 but higher concentrations of caspase 9 were found in comparison to the HE group at the 2nd and 3rd stage of study. This may indicate that the cell defense mechanisms, i.e., anti-apoptotic processes, have been activated. Such a conjecture, however, requires further research including the measurement of the NF-κB transcription factor. The role of TNFα in the process of sarcopenia is yet to be fully understood. Nevertheless, most studies have already shown that increased levels of TNFα and the activation of apoptotic pathways may contribute to skeletal muscle wasting [73].

The aging process is associated with an increased number of apoptotic cells and the release of extracellular DNA fragments into the bloodstream. cfDNA is used as a potential biomarker of aging that reflects the systemic inflammation and cell death. Some studies have also shown that higher cfDNA concentrations may be associated with weakness and degenerative diseases of the muscles and neurons [20,81]. In our study, cfDNA was not affected by Tai-Chi training in contrast to the control group, whose cfDNA level increased after 4 months and then decreased significantly in comparison to the TC group. Changes in the concentration of cfDNA depend also on the intensity and duration of exercise. However, with proper regeneration, cfDNA returns to resting values [82]. Finally, in the 3rd stage of the study, compared to the baseline values, in both groups, no increased DNA fragmentation was observed, which may indicate that the apoptotic processes were not activated.

## 5. Conclusions

Our findings were based on a randomized, controlled study that demonstrated the positive effect of exercise related to lifestyle to reduce some symptoms of sarcopenia. Tai-Chi training has pro-health effects through the loss of fat content and the improvement of physical performance. Practicing Tai-Chi may initiate changes in apoptotic mechanisms, which could improve the skeletal muscle functioning, and in the longer term, health-related quality of life in older adults.

## Figures and Tables

**Table 1:** Sarcopenia cut-off points.

TEST	Cut-Off Point for Women	Cut-Off Points for Men	References
Sarcopenia cut-off points for low muscle quantity			[3,32,33]
ASM	<15 kg	<20 kg	
ASMI	<5.5 kg/m^2^	<7.0 kg/m^2^	
Sarcopenia cut-off points for low performance			[3,34,35]
Gait speed	≤0.8 m/s	≤0.8 m/s	
Sarcopenia cut-off points for low strength by grip strength	<16 kg	<27 kg	[3,36]

Abbreviations: ASM, appendicular skeletal muscle mass; ASMI, appendicular skeletal muscle mass index.

**Table 2:** Changes in anthropometric factors depending on BMI in both the TC and HE group.

	BMI ≥ 30		BMI 25–29.9		BMI < 25	
	Mean ± SD	TC vs. HE *p*-Value	Mean ± SD	TC vs. HE *p*-Value	Mean ± SD	TC vs. HE *p*-Value
Age [yr]		0.231		0.321		0.683
TC	73.6 ± 7.9		69.8 ± 4.5		70.5 ± 9.1	
HE	72.1 ± 4.6		71.6 ± 3.2		72.1 ± 4.6	
BMI [kg/m^2^]		0.295		0.747		0.373
TC	31.4 ± 1.5		26.9 ± 1.3		22.8 ± 2.7	
HE	32.8 ± 2.1		27.1 ± 1.6		23.8 ± 1.4	
FM [kg]		0.42		0.988		0.272
TC	31.0 ± 4.8		24.3 ± 4.0		13.3 ± 5.9	
HE	33.5 ± 3.6		24.3 ± 2.5		19.9 ± 2.0	
FM [%]		0.209		0.944		0.214
TC	36.9 ± 1.9		36.0 ± 3.4		29.5 ± 7.4	
HE	41.7 ± 2.7		36.1 ± 2.5		33.1 ± 2.0	
MM [kg]		0.964		0.98		0.927
TC	44.7 ± 3.8		40.7 ± 3.8		38.0 ± 1.9	
HE	44.5 ± 4.8		40.7 ± 2.5		38.1 ± 3.1	
ASMI [kg/m^2^]		0.823		0.74		0.663
TC	7.6 ± 0.3		6.8 ± 0.3		6.3 ± 0.2	
HE	7.6 ± 0.5		6.8 ± 0.5		6.4 ± 0.5	
GRIP STRENGTH		0.828		0.345		0.244
Dominant hand [kg]						
TC	20.4 ± 5.8		21.9 ± 3.5		21.6 ± 3.4	
HE	19.5 ± 6.0		20.1 ± 5.4		17.9 ± 5.3	
Gait speed [m/s]		0.958		0.820		0.036
TC	1.2 ± 0.2		1.2 ± 0.1		1.5 ± 0.2	
HE	1.2 ± 0.3		1.2 ± 0.3		1.2 ± 0.2	
Sarcopenia [%]						
TC	0		0		0	
HE	0		0		0	

Abbreviations: TC: Tai-Chi training, HE: health education, BMI: body mass index, FM: fat mass, MM: muscle mass, ASMI: appendicular skeletal muscle mass index.

**Table 3:** Changes in body mass and body composition.

	1st Stage of Study		2nd Stage of Study		3rd Stage of Study		Comparison of Stages		
	Mean ± SD	TC vs. HE *p*-Value	Mean ± SD	TC vs. HE *p*-Value	Mean ± SD	TC vs. HE *p*-Value	1st vs. 2nd *p*-Value	1st vs. 3rd *p*-Value	2nd vs. 3rd *p*-Value
Weight [kg]		0.762		0.675		0.791			
TC	67.8 ± 9.7		67.9 ± 9.6		67.1 ± 9.3		0.985	0.08	0.056
HE	67.0 ± 8.8		66.8 ± 8.5		66.4 ± 8.1		0.773	0.217	0.572
Height [kg]		0.928		0.925		0.907			
TC	158.0 ± 5.9		157.9 ± 5.9		157.7 ± 6.0		0.920	0.278	0.477
HE	157.8 ± 3.6		157.8 ± 3.6		157.8 ± 3.5		0.980	0.981	0.981
BMI [kg/m^2^]		0.829		0.729		0.949			
TC	27.1 ± 3.2		27.2 ± 3.2		26.9 ± 3.2		0.951	0.089	0.025
HE	26.9 ± 3.6		26.8 ± 3.5		26.8 ± 3.2		0.859	0.754	0.980
FM [kg]		0.921		0.826		0.544			
TC	24.5 ± 6.1		24.6 ± 6.0		23.5 ± 5.8		0.960	0.003	0.001
HE	24.4 ± 5.5		24.2 ± 5.8		24.6 ± 5.9		0.967	0.909	*0.785*
FM [%]		0.778		0.716		0.324			
TC	35.7 ± 5.1		35.8 ± 4.9		34.6 ± 5.0		0.088	0.003 *	0.001 *
HE	36.0 ± 3.9		35.9 ± 4.5		36.3 ± 4.2		0.371	0.827	0.275
FFM [kg]		0.594		0.534		0.150			
TC	43.3 ± 4.3		43.3 ± 4.4		43.6 ± 4.3		0.995	0.568	0.515
HE	42.6 ± 4.0		42.5 ± 3.6		41.5 ± 5.1		0.986	0.212	0.278
MM [kg]		0.597		0.546		0.326			
TC	41.1 ± 4.1		41.1 ± 4.2		41.3 ± 4.1		0.997	0.562	0.518
HE	40.4 ± 3.8		40.4 ± 3.5		40.2 ± 3.6		0.946	0.542	0.739
RH MM [kg]		0.973		0.731		0.257			
TC	2.07 ± 0.24		2.10 ± 0.24		2.10 ± 0.25		0.393	0.264	0.963
HE	2.07 ± 0.24		2.07 ± 0.21		2.02 ± 0.20		1.000	0.076	0.076
LH MM [kg]		0.414		0.597		0.363			
TC	2.14 ± 0.29		2.11 ± 0.27		2.12 ± 0.27		0.477	0.683	0.940
HE	2.08 ± 0.24		2.07 ± 0.23		2.05 ± 0.22		0.969	0.463	0.609
RL MM [kg]		0.822		0.991		0.433			
TC	6.47 ± 0.58		6.53 ± 0.63		6.60 ± 0.65		0.420	0.037	0.281
HE	6.42 ± 0.74		6.52 ± 0.61		6.45 ± 0.59		0.287	0.909	0.511
LL MM [kg]		0.864		0.906		0.503			
TC	6.43 ± 0.57		6.47 ± 0.62		6.55 ± 0.60		0.514	0.042	0.155
HE	6.40 ± 0.58		6.45 ± 0.55		6.42 ± 0.59		0.482	0.858	0.802

Abbreviations: TC: Tai-Chi training, HE: health education, BMI: body mass index, FM: fat mass, FFM: fat-free mass, MM: muscle mass, RH MM: right hand muscle mass, LH MM: left hand muscle mass, RL MM: right leg muscle mass, LL MM: left leg muscle mass. * statistically significant interaction between group (TC vs. HE) and compared stages.

**Table 4:** Assessment of sarcopenia.

Assessment of Sarcopenia		1st Stage of Study		2nd Stage of Study		3rd Stage of Study		Comparison of Stages		
		Mean ± SD	TC vs. HE *p*-Value	Mean ± SD	TC vs. HE *p*-Value	Mean ± SD	TC vs. HE *p*-Value	1st vs. 2nd *p*-Value	1st vs. 3rd *p*-Value	2nd vs. 3rd *p*-Value
MUSCLE QUANTITY	ASM [kg]		0.773		0.569		0.406			
	TC	17.1 ± 1.6		17.2 ± 1.7		17.4 ± 1.7		0.634	0.047	0.284
	HE	17.0 ± 1.7		16.9 ± 1.8		17.0 ± 1.6		0.968	0.999	0.977
	ASMI [kg/m2]		0.795		0.607		0.405			
	TC	6.9 ± 0.5		6.9 ± 0.6		7.0 ± 0.6		0.634	0.044	0.27
	HE	6.8 ± 0.7		6.8 ± 0.7		6.8 ± 0.6		0.980	0.999	0.983
PHYSICAL PERFORMANCE	6MWT [m]		0.300		0.434		0.167			
	TC	450.9 ± 68.7		482.8 ± 62.9		488.1 ± 63.4		*0.002*	0.0004	0.812
	HE	426.9 ± 82.6		466.7 ± 72.6		459.3 ± 72.3		*0.003*	0.003	0.697
	Gait speed [m/s]		0.300		0.434		0.167			
	TC	1.25 ± 0.19		1.34 ± 0.17		1.36 ± 0.18		*0.002*	0.0004	0.812
	HE	1.19 ± 0.23		1.3 ± 0.2		1.28 ± 0.2		*0.0003*	0.003	0.697
GRIP STRENGTH	Dominant hand [kg]		0.098		0.037		0.436			
	TC	21.5 ± 3.9		22.1 ± 4.3		20.7 ± 4.4		0.613	0.211	0.003
	HE	19.2 ± 5.3		18.8 ± 5.5		19.4 ± 6.3		0.797	0.918	0.557
	Non-dominant hand [kg]		0.490		0.117		0.220			
	TC	18.4 ± 4.1		19.1 ± 3.8		18.7 ± 4.7		0.535	0.684	0.505
	HE	17.4 ± 4.9		16.9 ± 5.4		16.8 ± 5.1		0.531	0.460	0.993

Abbreviations: TC: Tai-Chi training, HE: health education, ASM: appendicular skeletal muscle mass, ASMI: appendicular skeletal muscle mass index, 6MWT: the 6-min walk test.

**Table 5:** Hematological variables, lipoprotein-lipid profile, glucose and C-reactive protein levels.

	1st Stage of Study		2nd Stage of Study		3rd Stage of Study		Comparison of Stages		
	Mean ± SD	TC vs. HE *p*-Value	Mean ± SD	TC vs. HE *p*-Value	Mean ± SD	TC vs. HE *p*-Value	1st vs. 2nd *p*-Value	1st vs. 3rd *p*-Value	2nd vs. 3rd *p*-Value
WBC [10^3^/µL]		0.519		0.226		0.178			
TC	6.7 ± 2.1		6.5 ± 1.6		6.7 ± 1.7		0.513	0.984	0.765
HE	6.3 ± 1.9		5.9 ± 1.8		5.6 ± 1.6		0.007	0.018	0.789
RBC [10^6^/µL]		0.002		0.038		0.002			
TC	4.9 ± 0.3		4.9 ± 0.3		4.9 ± 0.7		0.651	0.812	0.301
HE	4.7 ± 0.2		4.7 ± 0.3		4.4 ± 0.3		0.952	0.0005	0.0003
HB [g/dL]		0.038		0.119		0.447			
TC	14.0 ± 0.8		13.8 ± 0.7		13.6 ± 1.6		0.275	0.346	0.617
HE	13.5 ± 0.8		13.4 ± 0.8		13.6 ± 0.9		0.839	0.795	0.453
HCT [%]		0.051		0.465		0.813			
TC	39.6 ± 2.2		39.3 ± 2.5		41.0 ± 5.4		0.923	0.024	0.009
HE	38.2 ± 2.2		38.7 ± 2.4		41.3 ± 2.8		0.451	0.0001	0.0001
PLT [10^3^/µL]		0.770		0.252		0.099			
TC	264.9 ± 64.3		252.5 ± 82.9		279.5 ± 62.8		0.670	0.025	0.039
HE	273.6 ± 67.1		281.5 ± 71.6		241.8 ± 74.3		0.346	0.005	0.005
TChol [mg/dL]		0.335		0.736		0.303			
TC	222.9 ± 43.0		218.3 ± 43.6		232.7 ± 33.0		0.767	0.316	0.089
HE	235.3 ± 40.9		222.5 ± 36.2		242.9 ± 32.1		0.608	0.258	0.038
TG [mg/dL]		0.916		0.220		0.555			
TC	136.2 ± 23.9		152.1 ± 39.4		146.1 ± 32.4		0.011	0.371	0.491
HE	142.7 ± 32.6		138.3 ± 27.8		141.9 ± 21.3		0.827	0.827	0.827
LDL [mg/dL]		0.123		0.212		0.717			
TC	80.3 ± 19.7		102.6 ± 32.0		94.7 ± 37.9		0.002	0.148	0.282
HE	90.4 ± 23.7		115.0 ± 30.1		98.4 ± 25.7		0.778	0.225	0.655
HDL [mg/dL]		0.031		0.368		0.289			
TC	77.4 ± 10.8		94.2 ± 13.7		73.1 ± 10.7		0.0001	0.268	0.0002
HE	86.9 ± 18.8		98.4 ± 16.1		68.1 ± 18.7		0.025	0.005	0.00006
non-HDL [mg/dL]		0.845		0.838		0.087			
TC	145.6 ± 48.0		124.1 ± 46.5		159.2 ± 35.9		0.019	0.177	0.0002
HE	148.4 ± 47.9		121.4 ± 37.4		178.0 ± 34.4		0.027	0.0004	0.0001
Glucose [mg/dL]		0.903		0.039		0.059			
TC	99.9 ± 12.1		97.1 ± 12.3		92.0 ± 10.4		0.201	0.007	0.061
HE	100.4 ± 16.8		90.6 ± 12.4		87.7 ± 12.4		0.164	0.001	0.827
hsCRP [mg/L]		0.760		0.196		0.159			
TC	2.0 ± 1.7		3.8 ± 2.9		2.9 ± 1.8		0.0001	0.0004	0.144
HE	1.7 ± 1.3		3.1 ± 2.9		2.3 ± 1.8		0.005	0.005	0.827

Abbreviations: TC: Tai-Chi training, HE: health education, WBC: white blood cells, RBC: red blood cells, HB: hemoglobin, HCT hematocrit, PLT: platelets, TChol: total cholesterol, TG: triglycerides, LDL: low-density lipoprotein, HDL: high-density lipoprotein, hsCRP: high sensitivity C-reactive protein.

**Table 6:** The levels of inflammatory and apoptotic mediators.

	1st Stage of Study		2nd Stage of Study		3rd Stage of Study		Comparison of Stages		
	Mean ± SD	TC vs. HE *p*-Value	Mean ± SD	TC vs. HE *p*-Value	Mean ± SD	TC vs. HE *p*-Value	1st vs. 2nd *p*-Value	1st vs. 3rd *p*-Value	2nd vs. 3rd *p*-Value
TNFα [pg/mL]		0.949		0.307		0.159			
TC	0.8 ± 0.4		0.9 ± 0.4		3.0 ± 1.8		0.144	0.0001	0.0004
HE	0.8 ± 0.4		0.8 ± 0.3		2.3 ± 1.8		0.275	0.016	0.016
TNFRI [pg/mL]		0.279		0.869		0.459			
TC	159.6 ± 31.4		167.1 ± 37.2		163.8 ± 38.0		0.022	0.297	0.297
HE	148.2 ± 27.7		166.0 ± 35.6		147.3 ± 2.2		0.189	0.998	0.208
TNFRII [pg/mL]		0.790		0.597		0.354			
TC	336.6 ± 50.4		366.9 ± 68.1		409.2 ± 72.3		0.018	0.0001	0.002
HE	331.4 ± 50.7		357.4 ± 73.5		384.1 ± 56.5		0.064	0.0001	0.002
Cas 8 [ng/mL]		0.464		0.02		0.002			
TC	0.97 ± 0.27		0.08 ± 0.05		0.34 ± 0.05		0.000	0.0001	0.0001
HE	0.93 ± 0.13		0.12 ± 0.9		0.39 ± 0.6		0.0001	0.0001	0.0001
Cas 9 [ng/mL]		0.166		0.549		0.627			
TC	43.0 ± 17.3		4.9 ± 1.3		1.0 ± 2.0		0.0001	0.0001	0.0001
HE	35.7 ± 9.0		4.7 ± 1.2		0.6 ±0.3		0.0002	0.0002	0.047
cfDNA [ng/mL]		0.339		0.703		0.003			
TC	632.0 ± 116.4		663.5 ± 60.0		635.5 ± 74.4		0.263	0.935	0.440
HE	608.8 ± 74.3		687.6 ± 89.8		573.8 ± 67.1		0.016	0.827	0.0002

Abbreviations: TC: Tai-Chi training, HE: health education, TNFα: tumor necrosis factor α, TNFRI: soluble tumor necrosis factor receptor I, TNFRII: soluble tumor necrosis factor receptor II, Cas 8: caspase 8, Cas 9: caspase 9, cfDNA: cell-free DNA.

## Data Availability

The data presented in this study are available on request from the corresponding author.
